# Identification and validation of differentially expressed genes for targeted therapy in NSCLC using integrated bioinformatics analysis

**DOI:** 10.3389/fonc.2023.1206768

**Published:** 2023-05-31

**Authors:** Reem Altaf, Umair Ilyas, Anmei Ma, Meiqi Shi

**Affiliations:** ^1^ Department of Pharmacy, Iqra University, Islamabad, Pakistan; ^2^ Department of Pharmaceutics, Riphah Institute of Pharmaceutical Sciences, Riphah International University, Islamabad, Pakistan; ^3^ Department of Medical Oncology, Jiangsu Cancer Hospital and Jiangsu Institute of Cancer Research and the Affiliated Cancer Hospital of Nanjing Medical University, Nanjing, China; ^4^ Department of Clinical Pharmacy, School of Basic Medicine and Clinical Pharmacy, China Pharmaceutical University, Nanjing, China

**Keywords:** NSCLC, mutational analysis, differentially expressed genes, microarray, bioinformatics

## Abstract

**Background:**

Despite the high prevalence of lung cancer, with a five-year survival rate of only 23%, the underlying molecular mechanisms of non-small cell lung cancer (NSCLC) remain unknown. There is a great need to identify reliable candidate biomarker genes for early diagnosis and targeted therapeutic strategies to prevent cancer progression.

**Methods:**

In this study, four datasets obtained from the Gene Expression Omnibus were evaluated for NSCLC- associated differentially expressed genes (DEGs) using bioinformatics analysis. About 10 common significant DEGs were shortlisted based on their p-value and FDR (*DOCK4, ID2, SASH1, NPR1, GJA4, TBX2, CD24, HBEGF, GATA3, and DDR1).* The expression of significant genes was validated using experimental data obtained from TCGA and the Human Protein Atlas database. The human proteomic data for post- translational modifications was used to interpret the mutations in these genes.

**Results:**

Validation of DEGs revealed a significant difference in the expression of hub genes in normal and tumor tissues. Mutation analysis revealed 22.69%, 48.95%, and 47.21% sequence predicted disordered regions of DOCK4, GJA4, and HBEGF, respectively. The gene-gene and drug-gene network analysis revealed important interactions between genes and chemicals suggesting they could act as probable drug targets. The system-level network showed important interactions between these genes, and the drug interaction network showed that these genes are affected by several types of chemicals that could serve as potential drug targets.

**Conclusions:**

The study demonstrates the importance of systemic genetics in identifying potential drug- targeted therapies for NSCLC. The integrative system- level approach should contribute to a better understanding of disease etiology and may accelerate drug discovery for many cancer types.

## Introduction

The increasing incidence of lung cancer has made it the leading cause of cancer death among all human carcinomas. Globally, more than one million people die from lung cancer every year. Lung cancer (LC) consists of two main subtypes, non-small-cell lung cancer (NSCLC) and small-cell lung cancer (SCLC). The chief pathological form of lung cancer is NSCLC, accounting for 80-85% of cases. It includes adenocarcinoma (LUAC) and squamous cell carcinoma (LUSC) (1). With the remarkable advancement of medical technology in recent years, there is still an overall 5-year survival rate of 10-15% with no positive prognosis for lung cancer (2). The reason may be partly due to the problems encountered in the early diagnosis of the disease, in addition to the ineffective pharmacological targets for NSCLC patients (3). Mostly, the diagnosis of NSCLC is made when the disease has reached a progressive stage (2, 4). Some of the important risk factors that have shown an association with NSCLC are tobacco and air pollution, occupational hazards, and dietary and genetic factors that also contribute to its occurrence (5, 6). Over the past two decades, the treatment options for NSCLC have advanced significantly, requiring the need for alternatives to conventional treatment approaches, for example, molecularly targeted therapies and immunotherapies (3, 6).

The c omputational biology and systems biology approaches have greatly facilitated the drug discovery process, which has substantially minimized the cost of drug development. Several drug targets have been identified in our previous studies for breast cancer (7–9), colorectal cancer, methicillin- resistant *Staphylococcus aureus* (10–12), type 2 diabetes mellitus (13), and hTERT inhibitors (14). There is a great role for genes in the diagnosis, treatment, and prognosis of NSCLC when compared to histological classification. One of the most powerful and reliable techniques to quantify the expression of all genes is RNA microarray analysis (7, 10, 13–15). G ene expression profiling in NSCLC has been extensively done using RNA microarrays; nevertheless, not all genes have been fully explored. D ifferential expression analysis has been widely used as a bioinformatics tool in oncology research in recent years. The differentially expressed genes (DEGs) can be used to explore major diagnoses and identify effective therapeutic approaches for NSCLC, which play a crucial role in the management of cancer occurrence and progression (16). One of the bases for identifying novel targets and the molecular mechanism of NSCLC is to understand the interactions among the identified DEGs, their important signaling pathways, and the proteins through which they interact and cross- talk. Therefore, exploring the existing database and validating the effective targets is beneficial for the early diagnosis and therapeutic approach of NSCLC.

In this study, four microarray gene datasets (GSE1987, GSE17073, GSE 54495, and GSE118370) from the Gene Expression Omnibus (GEO) were accessed and analyzed. DEGs were analyzed between NSCLC tissue and normal lung tissue. Moreover, gene ontology, enrichment, and protein-protein interaction network analysis were performed to clarify the molecular mechanisms of the development and progression of NSCLC. Microarray technology assists in identifying unusual alterations in genome expression analysis. The identified DEGs may have the potential for future targeted therapy, providing better gene selection and serving as candidate biomarkers for NSCLC. This study also identified the genetic variants of NSCLC and their causes, which may help modify therapeutic strategies. 

## Materials and methods

### Processing of microarray datasets

The Gene Expression Omnibus database (GEO) is a public functional database for high- throughput screening of gene expression data, microarray data, and gene chips. In this study, we recovered genome expression datasets from GEO (Affymetrix Human Genome U133A Plus 2.0 Array, Affymetrix Human Genome U95A Array, and Affymetrix Human Genome U133 Plus 2.0 Array) [GSE1987, GSE17073, GSE 54495, GSE118370]. The GSE1987, GSE17073, GSE 54495, and GSE118370 datasets contain 28, two, 17, and six NSCLC tissue samples and 9, 10, 13, and six non-cancer tissue samples, respectively (**Table 1**). The software tools used in this study are listed in **Supplementary Table 1**.

**Table 1:** List of genome expression datasets analyzed in this study.

S. no	Geo ID	Sample count (case: control)	Platform used	Tissues
1.	GSE1987	9:28	GPL91[HG_U95A] Affymetrix Human Genome U95A Array	Lung tissue
2.	GSE17073	10:2	GPL96 [HG-U133A] Affymetrix Human Genome U133A Array	Lung epithelial cells
3.	GSE118370	6:6	GPL570 [HG-U133_Plus_2] Affymetrix Human Genome U133 Plus 2.0 Array	Lung tissue
4.	GSE54495	13:17	GPL570 [HG-U133_Plus_2] Affymetrix Human Genome U133 Plus 2.0 Array	Lung epithelial cells

### Raw data preprocessing, screening, and integration of DEGs

The differentially expressed genes were analyzed using GEO2R (http://www.ncbi.nlm.gov/geo2r), an interactive network tool for screening between NSCLC and non-cancer samples. The tool helps to compare and analyze two or more datasets under experimental conditions. The significant DEGs were detected using the adjusted p-values and the Benjamini- Hochberg false discovery rate. The probe sets with no gene names or genes with multiple probe sets were removed from the study. The upregulated genes were identified using cut- off values of P<0.05 and logFC >1, while the downregulated genes were identified with logFC <1.

### Disease gene curation of DEGs

The c uration of significant DEGs was performed with the help of the Comparative Toxicogenomics Database (CTD), the Online Mendelian Inheritance in Man (OMIM), PubMed, and MeSH databases in order to curate their role in NSCLC. The DAVID database was used to retrieve the gene symbol, name, and UniProt ID of the identified DEGs.

### Gene enrichment analysis

To identify the functional annotation and gene ontology of the shortlisted DEGs, the DAVID tool was used for functional enrichment analysis for their p- and FDR values. The biological function, transcription factors, and clinical phenotypes of NSCLC-related DEGs were analyzed using the FunRich tool.

### Mutation analysis

The genotype-phenotype association helps in decoding the genetic variations, which aids in understanding the mutations arising from cancer and the inherited disease- related processes. Several single nucleotide variants (SNVs) are part of the human genome and are involved in the progression of the disease. The missense SNVs present at the PTM (post-translation modification) protein sites are associated with disease progression due to the substitution of approximately 21% of amino acids. This chemical modification of the amino acid ultimately alters the function of the protein (17). The online web tool ActiveDriverDB database was used to analyze the mutations associated with differentially expressed genes (17). The overall visual summary of the position, frequency, and functional significance of the mutations in the DEGs was provided by the needle plot mutation analysis. We observed the predicted disordered region and the PTM sites for all mutations in the protein sequence. The position of the gene sequence was observed by placing the pin along the protein. The figure legends explain the related mutational effects and PTM sites.

### Protein-protein interaction

The protein-protein network analysis was performed to study the interaction of each protein with one or more genes associated with its molecular functions (18). The network reveals the altered activity of these genes in normal or pathological conditions. The potential NSCLC- related gene signatures associated with other genes whose dysfunction results in the disease state were identified through this network. The STRING database was used to analyze the protein-protein interactions of the cDNA dataset DEGs (19). The high confidence score interaction was used in this network analysis, having a score of 0.9-1. The target genes identified by this network were then studied for their role in NSCLC using Cancer GeneticsWeb, the National Cancer Institute, and the OMIM databases. Cytoscape (version 3.9.1) (20) was used to visualize the molecular network and Network Analyzer was used to calculate the topological properties of the networks. The nodes in the network categorized the degree of annotation between genes and diseases.

### Identification and validation of shortlisted DEGs

T o validate the expression pattern of identified DEGs in normal and tumor tissues, the GEPIA database was utilized (21). GEPIA (Gene Expression Profiling Interactive Analysis) is helpful in differential expression analysis, patient survival analysis, correlation and profiling plotting, and several other key interactive and customizable features. G ene expression plots in GEPIA are based on TCGA clinical annotations. The Human Protein Atlas database was used to validate the translational levels of the identified oncogenes. V alidation was performed using immunohistopathologic sections between the normal and OSCC samples (https://www.proteinatlas.org/).

### Toxicogenomic analysis

The comparative toxicogenomic database (CTD) was used to perform the toxicogenomic analysis. The CTD helps in retrieving the exposome data, which explores the chemical-genome to phenome association. The analysis investigates the mechanism of functional pathway signaling toward the progression of the disease. The chemical-gene and disease interactions are inferred, revealing the particular expression of the gene and its association with disease (22).

### miRNA prediction analysis

microRNAs (miRNAs) are small non- coding RNAs that act as post-translational regulators that influence the genes involved in biological signaling pathways. In order to study the gene etiology, the expression and functional role of miRNA play a significant role (23). The disease- specific functional and molecular annotation of DEGs can be investigated by predicting the miRNA target (24). The miRDB online database was used to predict the NSCLC- related DEG miRNA targets for functional target prediction. The prediction data includes several important descriptions, such as 3′-UTR region length, miRNA-candidate target pairs along with target prediction scores, 3′-UTR sequences, miRNA seed binding sites, miRNA target sequences, etc. A score >80 was considered reliable, and the miRNA target was ranked (25, 26). The miRNA targets were analyzed for their site of expression and the biological pathways in which they are commonly used, with the help of the FunRich tool.

### Drug-gene network

The drug -gene network analysis was performed to correlate the shortlisted DEGs with the chemicals/drugs that affect the activity of these genes. The CTD database was used to screen the chemical and disease relationships with default parameters. A direct link between the DEGs and anticancer drugs was developed. The FDA approval status of the identified drugs was verified with the Drug Bank database.

## Results

### Differential expression analysis and identification of DEGs

**Figure 1:**
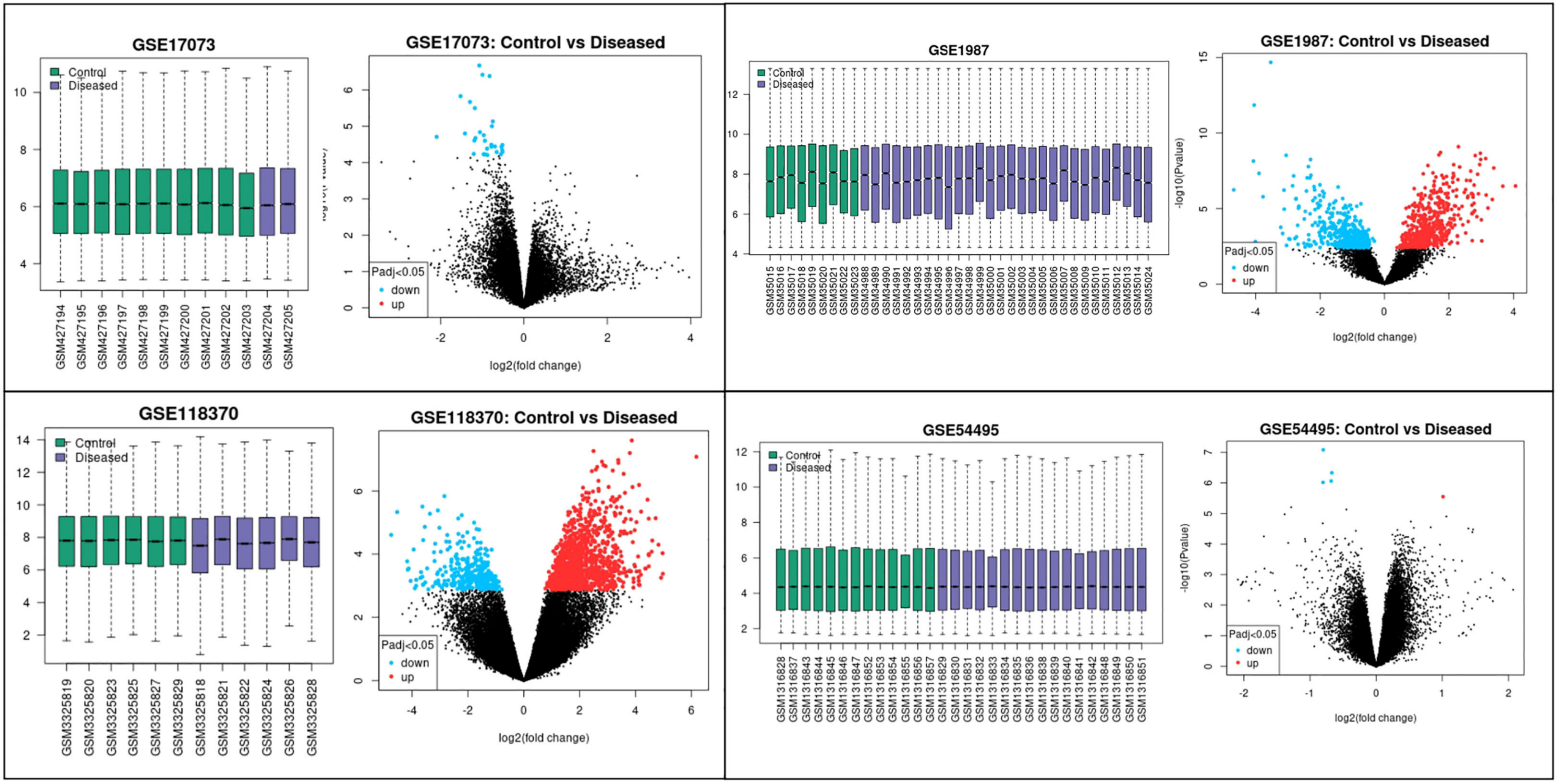
Data accessed from GEO were analyzed in GEO2R. The box plot generated by R represents the normalization of the data obtained after the log transformation. The volcano plot visualizes the DEGs by displaying the statistical significance –log10 p- value versus the log2 fold change. The significantly upregulated and downregulated differentially expressed genes in NSCLC are the highlighted genes in the four datasets. The blue color represents the downregulated genes, while the red color represents the upregulated genes in NSCLC.

**Figure 2:**
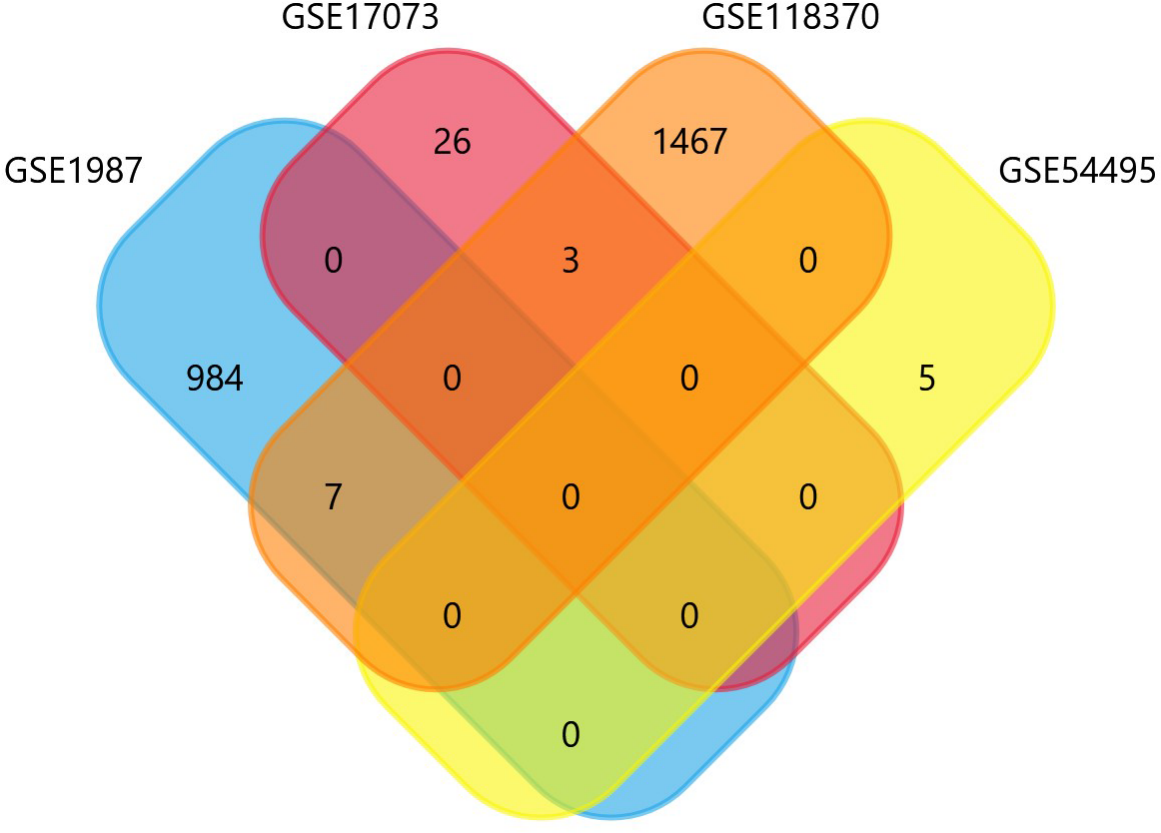
Identification of common NSCLC- related oncogenes using the Venn diagram.

The four datasets GSE17073, GSE1987, GSE54495, and GSE118370 were used to screen and identify the DEGs after obtaining the microarray normalization results. The dataset GSE17073 contained 30 significant DEGs, GSE1987 contained 508 significant DEGs, GSE54459 contained five significant DEGs, and GSE118370 contained 707 significant DEGs. The volcano plot and the mean difference plot of DEGs from different datasets revealed the significant genes that were upregulated and downregulated. The blue color shows the down regulated genes, while the red indicates the upregulated genes (**Figure 1**). In GSE1987, GSE17073, GSE54495, and GSE118370, 403, zero, one, and 354 genes are upregulated, respectively, while 405, 30, four, and 353 DEGs are downregulated. In the Venn diagram, the overlap between the datasets was observed, which showed an overlap of 10 significant DEGs with a p-value of <0.05 and logFC <1 for downregulated genes and logFC >1 for upregulated genes (**Figure 2**). **Table 2** shows the expression profiles of the microarray datasets.

**Table 2:** Expression profiles of the microarray datasets.

AFFYMETRIX_ 3PRIME_ IVT_ID	Gene name	logFC	t	p-value	adj. p-	Val B	Aberration
205003_at	DOCK4	1.461537	4.333	8.65E-04	0.039831	-0.4456	Upregulated
41644_at	SASH1	9.99E-01	3.83	4.61E-04	1.28E-02	-0.2121	Downregulated
201565_s_at	ID2	1.651813	6.222	3.52E-05	0.008697	2.6173	Upregulated
40687_at	GJA4	1.54	4.74	2.99E-05	1.75E-03	2.3544	Upregulated
40560_at	TBX2	1.52	4.35	9.80E-05	4.27E-03	1.2370	Upregulated
38037_at	HBEGF	1.34	3.9	3.84E-04	1.13E-02	-0.0411	Upregulated
32625_at	NPR1	1.49	3.68	7.16E-04	1.66E-02	-0.6214	Upregulated
209604_s_at	GATA3	1.461537	4.333	8.65E-04	0.039831	-0.4456	Upregulated
266_s_at	CD24	-2.06	-5.13	8.84E-06	7.14E-04	3.5054	Downregulated
1007_s_at	DDR1	-9.14E-01	-3.71	6.64E-04	1.58E-02	-0.5521	Downregulated

### Curation of DEGs

**Figure 3:**
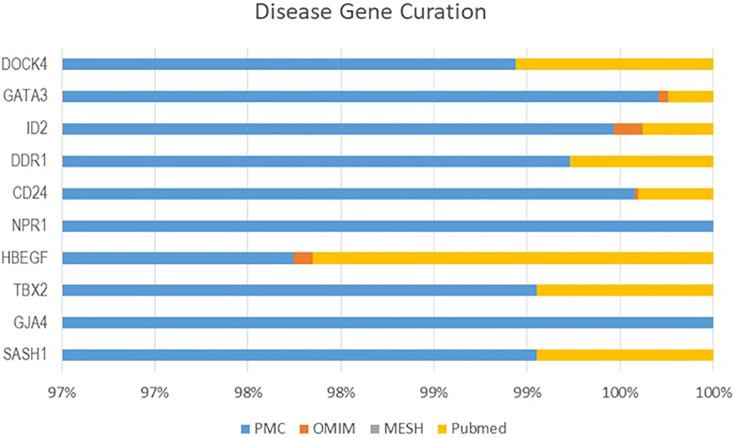
The NSCLC-related oncogenes were curated using CTD, MeSH, OMIM, and PubMed.

From four datasets, we found 10 common DEGs whose gene symbols and biological annotations were retrieved using the David tool. D isease-gene curation was performed by text mining with the help of PubMed, PMC, PMIM, and MeSH (**Supplementary Table 1**). It was observed that the DEGs *DOCK4, ID2, GATA3, SASH1, GJA4, TBX2, HBEF, NPR1, CD24*, and *DDR1* were the most curated terms in the databases. The mapping of these genes by cancer genetics was performed to further analyze their role in carcinogenesis (**Figure 3**).

### Enrichment analysis of DEGs

**Figure 4:**
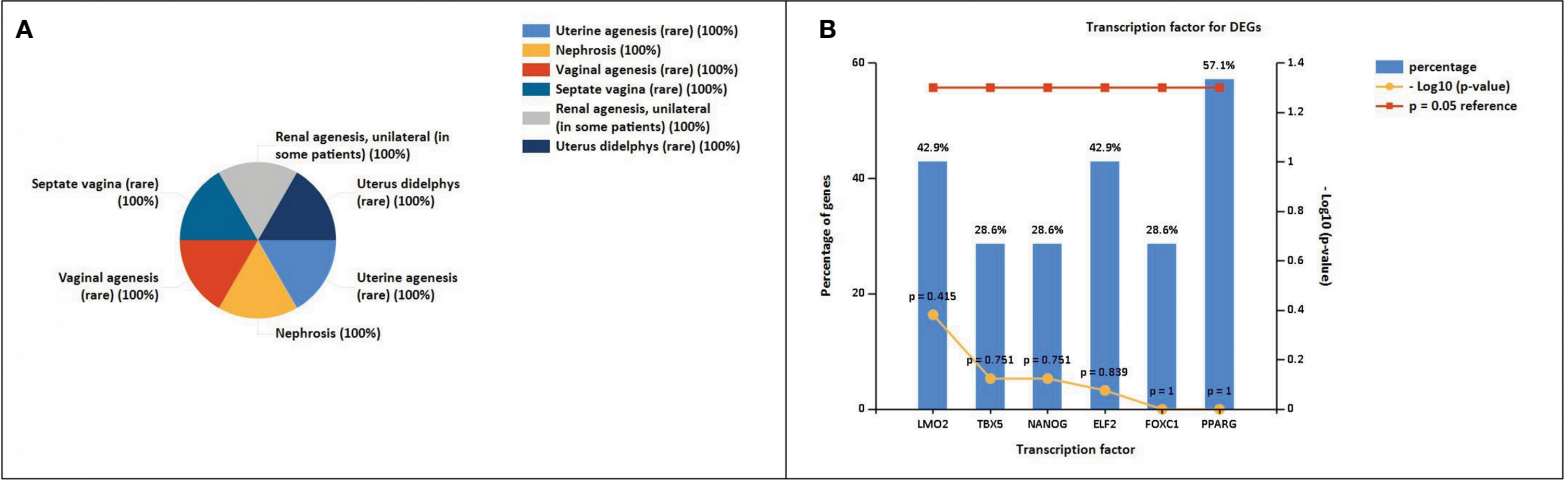
**(A)** Clinical phenotypes associated with NSCLC-related DEGs using the FunRich tool. **(B)** Transcription factors identified for NSCLC DEGs using the FunRich tool (p<0.05).

The enrichment analysis of DEGs showed that these genes were significantly linked to the regulation of epithelial cell differentiation, ear development, mammary gland alveolus development, regulation of cardiac contraction, cellular senescence, negative regulation of transcription, DNA-templated synthesis, positive regulation of endothelial cell migration, positive regulation of smooth muscle cell proliferation, response to estrogen, and cell chemotaxis (**Table 3**). The clinical phenotypes associated with the *GATA3* gene were n ephrosis and renal agenesis. Vaginal agenesis, septate vagina, uterine agenesis, and uterus didelphys are rarely associated with this gene (**Figure 4**). The transcriptome analysis revealed expressive transcription factors encoded by these genes, such as *LMO2, ELF2, TBX5, PPARG*, and *FOXC1* (**Figure 4**).

**Table 3:** Functional annotation and gene ontology of DEGs.

Term	p-value	Fold enrichment	FDR
Regulation of epithelial cell differentiation	3.70E-03	482.7	6.50E-01
Ear development	4.70E-03	386.2	6.50E-01
Mammary gland alveolus development	8.40E-03	214.5	7.80E-01
Regulation of heart contraction	1.60E-02	113.6	9.20E-01
Cellular senescence	2.50E-02	71.5	9.20E-01
Negative regulation of transcription, DNA-templated	2.90E-02	9.8	9.20E-01
Positive regulation of endothelial cell migration	3.10E-02	56.8	9.20E-01
Positive regulation of smooth muscle cell proliferation	3.20E-02	55.2	9.20E-01
Response to estrogen	3.30E-02	54.4	9.20E-01
Cell chemotaxis	3.30E-02	54.4	9.20E-01
Positive regulation of protein kinase B signaling	5.90E-02	29.9	1.00E+00
Serine-threonine/tyrosine-protein kinase catalytic domain	6.00E-02	29	1.00E+00
Parathyroid hormone synthesis, secretion and action	6.30E-02	25.7	1.00E+00
Negative regulation of transcription from RNA polymerase II promoter	7.20E-02	6	1.00E+00
Cell proliferation	8.30E-02	20.8	1.00E+00
RNA polymerase II sequence-specific DNA binding transcription factor binding	8.70E-02	19.9	1.00E+00
DOMAIN: SH3	9.10E-02	19	1.00E+00
Receptor complex	9.10E-02	19	1.00E+00
Src homology-3 domain	9.20E-02	18.6	1.00E+00

### Mutational analysis

**Figure 5:**
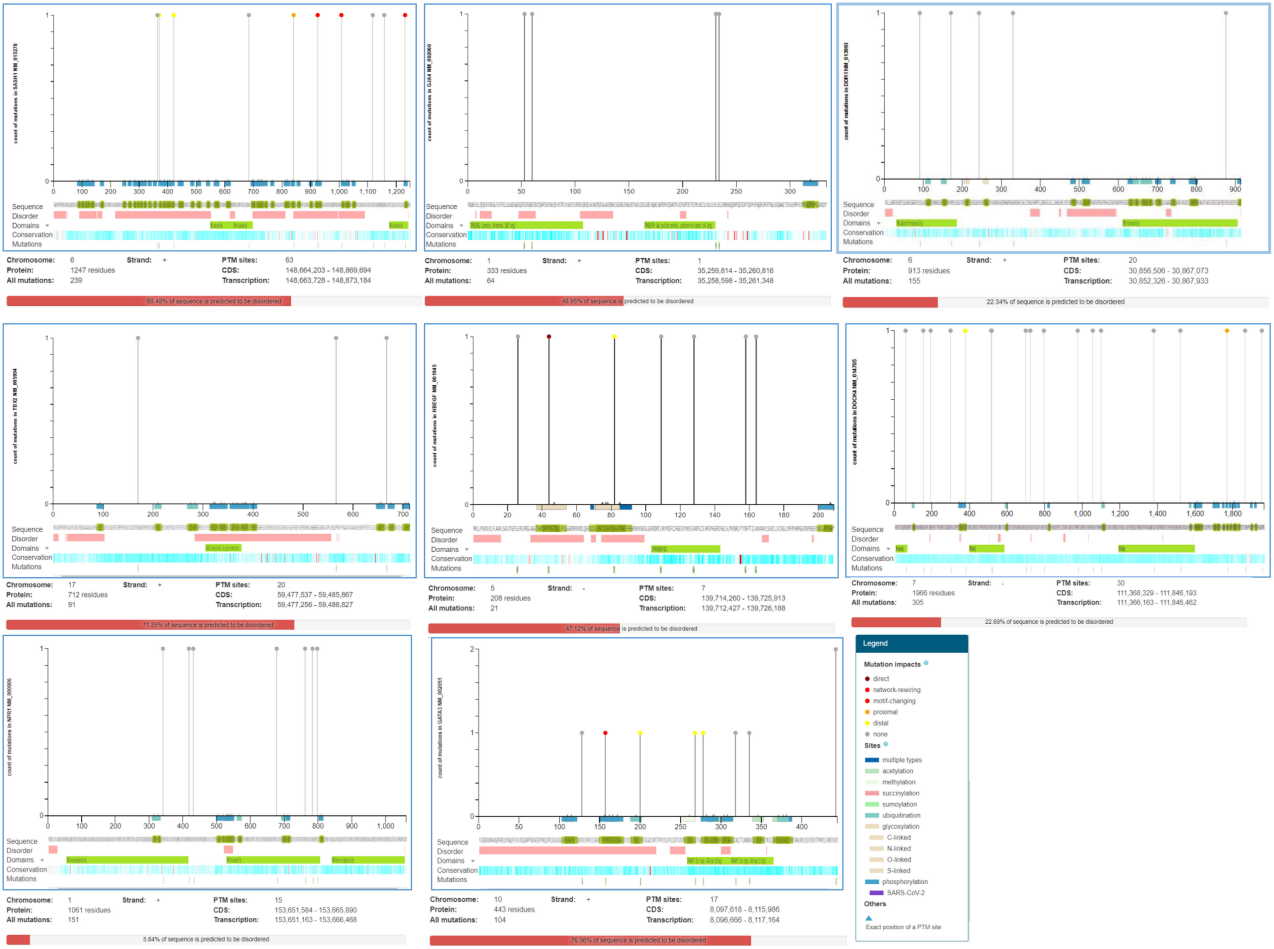
ActiveDriverDB database showing mutations impacting post-translational modification (PTM) sites in proteins. Needle plots indicate PTM site mutations in the identified DEGs. The x-axis indicates the position of the amino acid sequence, while the y-axis shows the number of mutation s. The shading of the x-axis reveals the type of PTM associated with the mutation site.

*Dock4* consists of 30 post-translational modification (PTM) sites having 305 recurrent cancer mutations on the 7- chromosome number negative strand encoding 1966 protein residues with 22.69% predicted disordered regions. The mutation visualization plot shows the *DOCK4* isoform mutation at position 378 with the reference amino acid residue V compared to the mutated amino acid residue L, which is enriched for phosphorylation-type mutations, showing the distal mutation PTM impact with the affected site. At position 1770, the L amino acid residue that was enriched with the phosphorylation-type mutation showed proximal mutation. The PTM impact with affected sites were 1769S. *SASH1* showed a 68.48% predicted disordered region with 239 mutations observed on chromosome number 6 on the positive strand. 247 protein residues were encoded and 63 PTM sites were observed. At positions 370 and 421, the reference G and R amino acid residues were compared with W and C mutated amino acid residues showing distal PTM mutation impact with affected sites 374S and 417S, respectively. The affected sites at positions 925, 1231, and 1008 have P, L, and S residue sites enriched with a phosphorylation-type mutation affecting PTMs showing a network-rewiring motif gain mutation with mutated amino acid residue R, respectively. A proximal PTM impact at the 837S and 839S affected sites at position 841 was observed with reference amino acid residue D compared to the mutated Y amino acid residue. Similarly, the mutational analysis of *GJA4* showed 48.95% of the sequence predicted for disease pathophysiology. A total of 64 mutations were found on the positive strand of chromosome number 1 for *GJA4*. The number of PTM sites was one, with 333 amino acid residues. In protein *HBEGF*, 21 mutations were observed on the negative strand of chromosome number 5 encoding 208 protein residues with 47.12% predicted disordered regions (**Figure 5**).

The mutational analysis of *NPR1* showed 15 PTM-affected sites with 151 mutations on the positive strand of chromosome number 1 encoding 1061 protein residues, representing 5.84% of predicted disordered regions. *GATA3* showed 76.98% disordered regions with 104 mutations on the positive strand of chromosome number 10 encoding 443 protein residues. In position 157, reference amino acid P showed network rewiring motif loss PTM impacts at affected sites 156S and 162S compared with mutated amino acid T. In positions 278, 268, and 200, reference amino acids G, A, and H and mutated amino acids S, E, and Q showed distal PTM impact enriched with phosphorylation, methylation, and ubiquitination-type mutations, respectively. Similarly, the number of PTM sites for *DDR1* was 20, with 913 protein residues having 22.34% of the predicted sequence for the disordered region. A total of 155 mutations were found on the positive strand of chromosome number 6 for *DDR1* (**Figure 5**). Eight isoforms of DDR1 were found (**Supplementary Table 2**).

### Protein -protein network analysis

**Figure 6:**
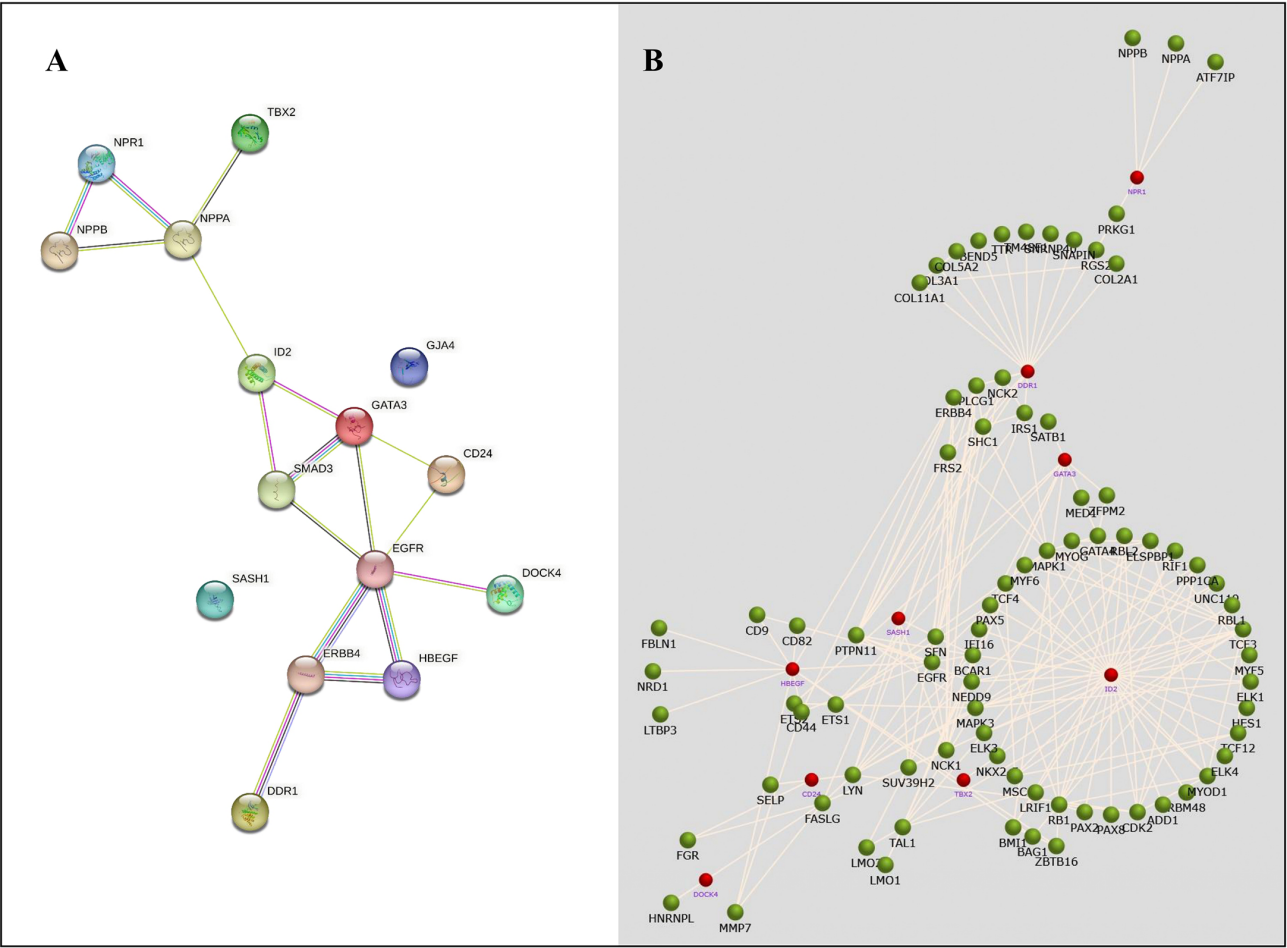
The protein-protein network analysis for identified NSCLC DEGs. **(A)** A network obtained from the STRING database shows a confidence score of 0.9. **(B)** A network obtained from the FunRich tool shows the association of several genes with NSCLC-related genes.

The STRING database was used to retrieve the related nodes and edges of all NSCLC- associated DEGs (**Figure 6**). The PPI enrichment p-value was 0.012 with 15 nodes and 17 edges. **Figure 6B** shows the upregulated and downregulated oncogenes and their associated genes, allowing us to evaluate their biological functions.

### Validation of shortlisted DEGs

**Figure 7:**
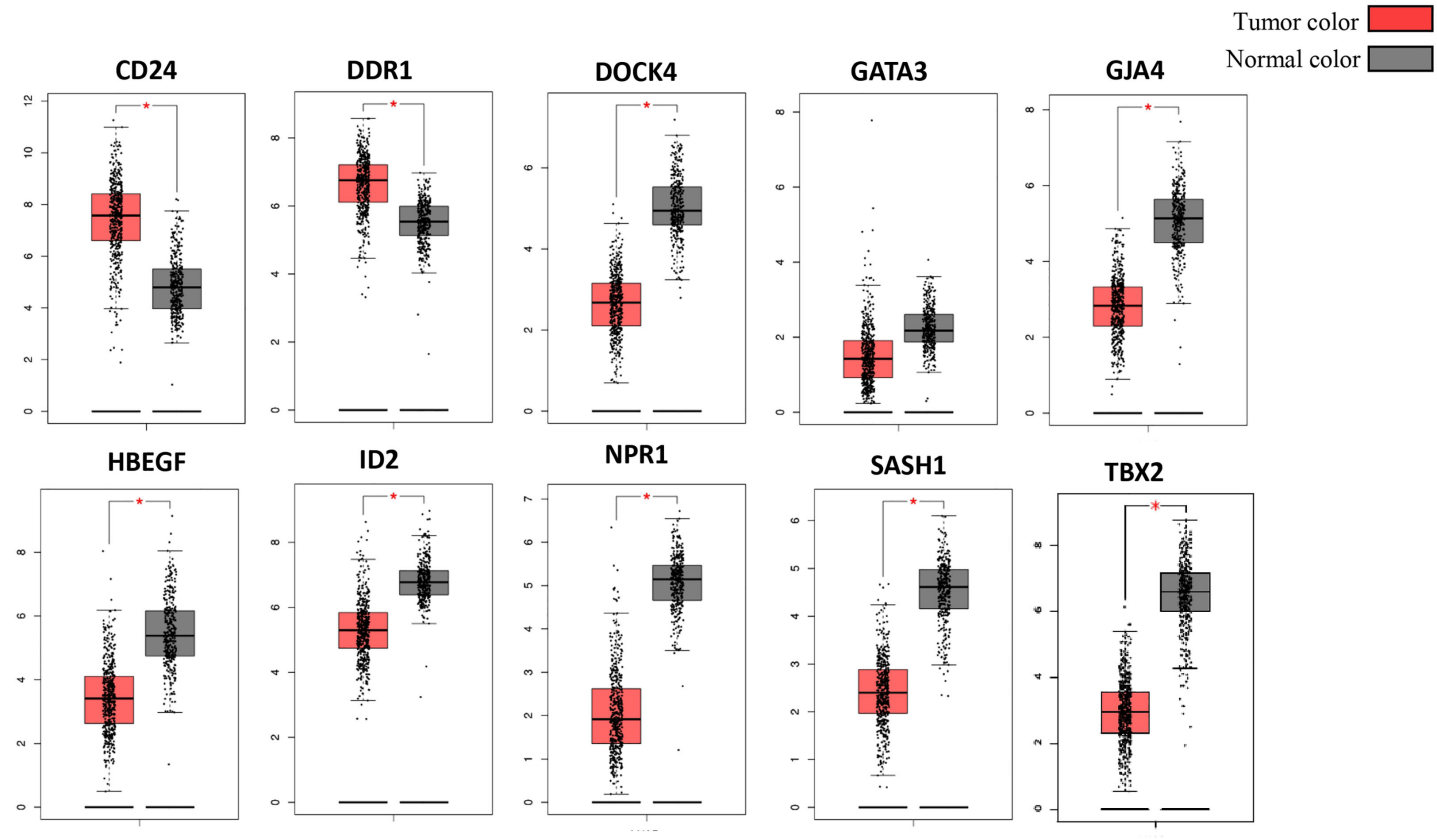
Validation of identified DEGs in the Cancer Genome Atlas (TCGA) database. The box plot shows the expression of genes in mRNA using data from the TCGA database in GEPIA. The method for differential analysis is one-way ANOVA, using disease state. The data was consistent with our study, and their p-values <0.05. The * indicates the difference in gene expression between normal and diseased tissues.

**Figure 8:**
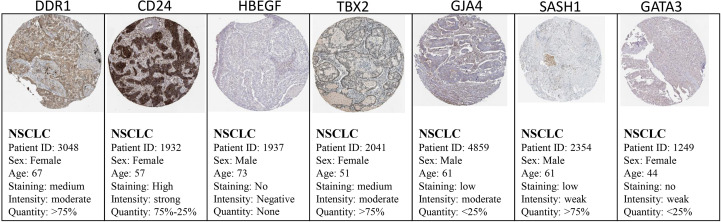
Validation of the identified DEGs at the translational level using the Human Protein Atlas database. The seed genes showed expression in the tissues of NSCLC patients.

The Cancer Genome Atlas (TCGA) and the GEPIA databases were employed to further validate our findings. The GEPIA NSCLC data showed that the expressions of these identified DEGs were significantly different between the normal and tumor tissues (**Figure 7**). The trend was the same as observed in our data, which is consistent with the GEO analysis. Moreover, the Human Protein Atlas database, which showed deregulation of the expression of these seven genes, was used to obtain their immunohistochemistry staining data (**Figure 8**). E xpression of the hub genes *HBEGF, GJA4, DDR1, CD24, TBX2, GATA3*, and *SASH1* did not appear to be prognostic in lung cancer. No pathological data were found for *DOCK4, NPR1*, or *ID2* genes.

### Toxicogenomic analysis

**Figure 9:**
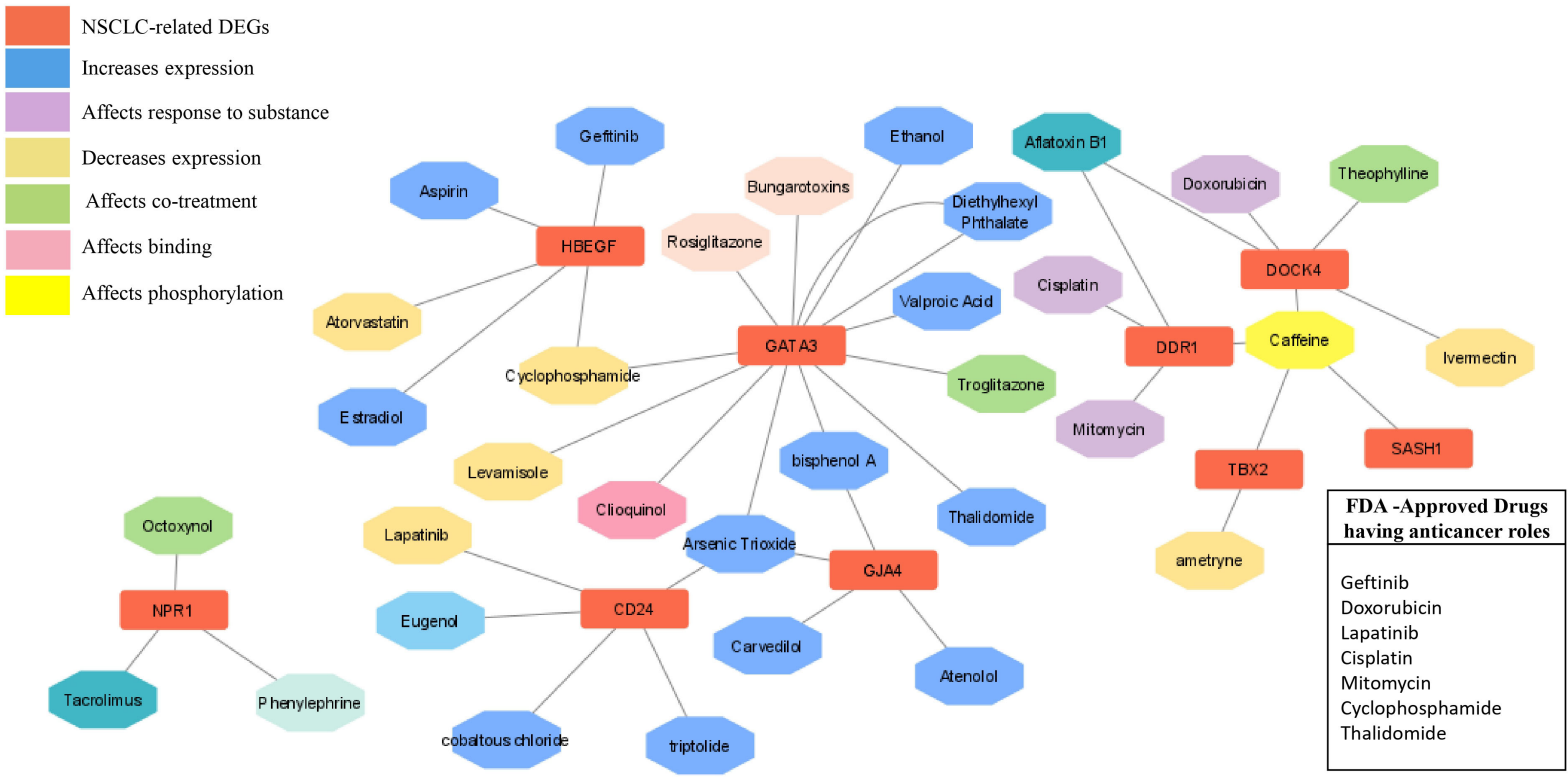
Drug- gene network analysis. The network shows the interaction of important drugs with the identified differentially expressed genes in NSCLC. The red color represents the NSCLC -related DEGs, and their interaction types, such as increased expression after responding to a substance, decreased expression, co-treatment, binding, and phosphorylation are represented by different colors.

The chemical-genotype-phenotype exposome data that may lead to disease progression were explored with the help of toxicogenomic analysis. The effect of different environmental chemicals on the activity and expression of NSCLC- associated DEGs was curated. The data revealed the effects of several chemicals on the increase or decrease of the expression of these NSCLC DEGs on gene activity at different levels (**Figure 9**). It was also revealed that the co-treatment expression leading to disease occurrence and the same chemical exposure showed different reactivity for different genes. For example, arsenic trioxide decreases the expression of *GJA4*, but it affects the binding of *GATA3*. The details of the effect of these chemicals on NSCLC genes are shown in **Table 4**.

**Table 4:** Interaction of different chemicals/drugs with the identified NSCLC DEGs.

Gene	Chemical	Interaction Actions
CD24	Arsenic Trioxide	Increases expression
CD24	Cobaltous chloride	Increases expression
CD24	Eugenol	Decreases expression
CD24	Lapatinib	Decreases expression
CD24	Triptolide	Increases expression
DDR1	Aflatoxin B1	Affects expression
DDR1	Caffeine	Decreases phosphorylation
DDR1	Cisplatin	Affects response to substance
DDR1	Mitomycin	Affects response to substance
DOCK4	Aflatoxin B1	Affects expression
DOCK4	Caffeine	Affects phosphorylation
DOCK4	Doxorubicin	Affects response to substance
DOCK4	Ivermectin	Decreases expression
DOCK4	Theophylline	Affects co-treatment
GATA3	Arsenic Trioxide	Affects binding, decreases reaction
GATA3	Bisphenol A	Increases expression
GATA3	Bungarotoxins	Decreases reaction, increases activity
GATA3	Clioquinol	Affects binding, decreases reaction
GATA3	Cyclophosphamide	Decreases expression
GATA3	Diethylhexyl Phthalate	Decreases reaction, increases expression
GATA3	Diethylhexyl Phthalate	Increases expression
GATA3	Ethanol	Increases expression
GATA3	Levamisole	Decreases expression, decreases reaction
GATA3	Rosiglitazone	Decreases reaction, increases degradation, increases ubiquitination
GATA3	Thalidomide	Increases expression
GATA3	Troglitazone	Affects co-treatment, increases expression
GATA3	Valproic Acid	Increases expression
GJA4	Arsenic Trioxide	Decreases expression
GJA4	Atenolol	Increases expression
GJA4	Bisphenol A	Increases expression
GJA4	Carvedilol	Increases expression
HBEGF	Aspirin	Decreases reaction, increases expression
HBEGF	Atorvastatin	Decreases expression
HBEGF	Cyclophosphamide	Decreases expression
HBEGF	Estradiol	Increases abundance, increases expression
HBEGF	Gefitinib	Decreases reaction, increases activity, increases expression, increases secretion
NPR1	Octoxynol	Affects co-treatment, increases activity
NPR1	Phenylephrine	Increases activity increases localization, increases reaction
NPR1	Tacrolimus	Affects expression, decreases reaction
SASH1	Caffeine	Affects phosphorylation
TBX2	Ametryne	Decreases expression
TBX2	Caffeine	Affects phosphorylation

### miRNA target prediction

**Figure 10:**
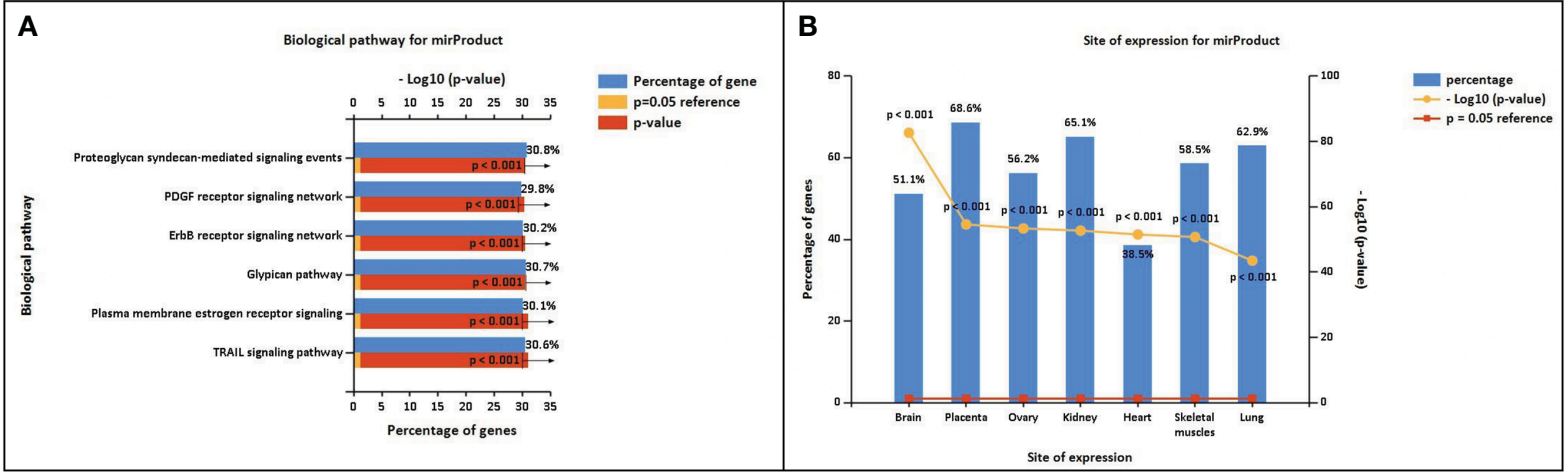
**(A)** Biological pathway associated with the miRNA predicted targets. **(B)** Site of expression of mirProduct (p<0.05) showing significant expression in the lungs.

The miRNA targets were predicted with the help of the miRDB database based on the algorithms. The miRNA targets such as hsa-miR-302c-5p, hsa-miR-4531, hsa-miR-11181-5p, hsa-miR-4476, hsa-miR-338-5p, hsa-miR-194-5p, hsa-miR-4279, hsa-miR-4742-3p, hsa-miR-4530, and hsa-miR-199a-5p were predicted for *DOCK4, SASH1, ID2, GJA4, TBX2, HBEGF, NPR1, GATA3, and CD24*, respectively. The onset and progression of the disease may be caused by the deregulation of these genes. **Table 5** shows the predicted scores, the total number of miRNA hits, and the seed location of the DEGs. The functional enrichment analysis of these miRNA targets was performed using the FunRich tool. The analysis revealed some important biological pathways associated with these targets, such as the PDGF receptor signaling network, the ErbB receptor signaling network, the glypican signaling pathway, the TRAIL signaling pathway, and the plasma membrane estrogen receptor signaling pathway (**Figure 10**). The sites of expression for these miRNA targets analyzed were brain (51.1%), placenta (68.6%), ovary (56.2%), kidney (65.1%), heart (38.5%), skeletal muscles (58.5%), and lung (62.9%), with p<0.001. 

**Table 5:** Predicted miRNA targets.

Gene symbol	Gene description	Target score	miRNA name	Total hits	miRNA sequence	Seed location	3′-UTR length
DOCK4	Dedicator of cytokinesis 4	100	hsa-miR-302c-5p	195	UUUAACAUGGGGGUACCUGCUG	182, 1270, 1653	2173
SASH1	SAM and SH3 domain containing 1	96	hsa-miR-4531	204	AUGGAGAAGGCUUCUGA	2352, 3248	3498
ID2	Inhibitor of DNA binding 2	95	hsa-miR-11181-5p	78	GUCUGACCAACCUCCUCCCGC	401	814
GJA4	Gap junction protein alpha 4	92	hsa-miR-4476	20	CAGGAAGGAUUUAGGGACAGGC	164, 481	620
TBX2	T-box 2	86	hsa-miR-338-5p	57	AACAAUAUCCUGGUGCUGAGUG	741	976
HBEGF	Heparin-binding EGF- like growth factor	99	hsa-miR-194-5p	133	UGUAACAGCAACUCCAUGUGGA	677, 1381	1479
NPR1	Natriuretic peptide receptor 1	84	hsa-miR-4279	34	CUCUCCUCCCGGCUUC	355	594
GATA3	GATA binding protein 3	97	hsa-miR-4742-3p	128	UCUGUAUUCUCCUUUGCCUGCAG	738, 1115	1178
CD24	CD24 molecule	96	hsa-miR-4530	94	CCCAGCAGGACGGGAGCG	217	1830
DDR1	Discoidin domain receptor tyrosine kinase 1	100	hsa-miR-199a-5p	89	CCCAGUGUUCAGACUACCUGUUC	1180, 1214, 1275, 1398	1735

### Drug-gene network analysis

In order to explore the available treatment options for NSCLC, a toxicogenomic analysis was performed. The anti-cancer drugs gefitinib, doxorubicin, lapatinib, cisplatin, mitomycin, cyclophosphamide, and thalidomide showed interactions with the hub genes using the CTD database. Several other FDA- approved drugs also showed interactions with the seed genes, such as theophylline, rosiglitazone, aspirin, estradiol, phenylephrine, atorvastatin, and valproic acid. These drugs may serve as novel targets for these genes in the treatment of NSCLC (**Figure 9**).

## Discussion

The study is based on the evaluation of the genetic expression of identified NSCLC DEGs and the understanding of the functional enrichment of their genetic variants. The differential expression analysis revealed 10 significant NSCLC- associated genes (*DOCK4, SASH1, ID2, GJA4, TBX2, HBEGF, NPR1, GATA3*, and *CD24)* and were considered the seed genes. The differential expression of the cDNA datasets between cases and controls was explored at the cellular level in lung tissues, and a possible association of these genes was observed in NSCLC cancer. Microarray studies can help in acquiring further information regarding the mechanisms of human genetic disorders.

The protein-protein network analysis revealed that these hub genes have an important association with the disease. These genes showed interaction with several important genes that have a role in NSCLC development, such as, *EGFR* (27), *ERBB4* (28–30), *SMAD3* (31–33), and *NPPA* (34), showing potential cross-talk between these genes in the progression of NSCLC. The hub genes showed important roles in TGF-beta, Rap1, ErbB, and GnRH signaling pathways and hematopoietic cell lineages. T ranscriptome analysis revealed expressive transcription factors encoded by these genes, such as *LMO2, ELF2, TBX5, PPARG*, and *FOXC1*. The dysregulation of miRNAs in these genes leads to disease progression and onset, as they are involved in the regulation of post-transcriptional and translational events (35, 36). Therefore, target prediction of miRNAs to aid in functional annotation is crucial (37, 38).

The expression profiling was validated using different databases, such as GEPIA and the Human Protein Atlas, which confirmed the expression of these genes in the diseased state by experimental analysis. The box plot obtained from GEPIA showed an obvious difference in the expression of these genes in the control and disease states. The large-scale characterization of human genomes has become possible through DNA sequencing studies, with different types of genetic variants, such as single nucleotide variants (SNVs) and copy number variations being revealed using this approach. The major challenge in current biomedical research is to determine the association of genotype with phenotypic characteristics, the molecular mechanisms underlying a disease state, the mutations underlying a cancerous state, or any disease variants (39, 40). Several projects are now available that have a large catalog of genetic variants, such as the Cancer Genome Atlas (TCGA), the International Cancer Genome Consortium (ICGC), and others that provide information about thousands of individual and tumor genomes. Post -translational modification (PTM) involves molecular switches of more than 400 amino acid chemical modifications that expand the functional repositories of proteins (31, 41). Almost 400,000 human protein sites are experimentally determined to act as PTM sites, which include phosphorylation, acetylation, ubiquitination, and methylation (42, 43). These PTM sites are helpful in personalized therapies for cancer and are good drug target sites as they aid in the interpretation of genetic variants, the association of genotype and phenotype, and the underlying molecular mechanisms of disease (44, 45). Our study revealed the PTM sites of the hub genes that might be involved in the progression of NSCLC. Moreover, the immunohistochemistry data also showed the expression of these genes in the pathological condition of lung cancer. *SASH1* (SAM and SH3 domain-containing protein 1) has a major role in cellular processes such as apoptosis and cellular proliferation. It acts as a tumor suppressor protein. D ifferential expression analysis revealed that SASH1 is downregulated in NSCLC, which may be a factor leading to cancer progression. Burgess et al. (46) studied the association of low *SASH1* mRNA expression with poor survival in adenocarcinoma. Their results showed that the compounds that increase the expression level of *SASH1* could be used as a novel approach to treating NSCLC, which warrants further studies (46, 47). The expression of HBEGF and its role in lung cancer have been studied by several scientists. *HBEGF* (heparin-binding EGF-like growth factor) belongs to the EGF family of growth factors and acts as an EGFR ligand. It is more potent than EGF in inducing cellular proliferation and migration. HBEGF has been shown to be upregulated in several cancers, including lung cancer. The gene generates signals for differentiation, migration, proliferation, and cell survival by binding to and over- activating the EGFR pathway (38, 48, 49). Our analysis also revealed overexpression of this gene in NSCLC, which could serve as a potential therapeutic target for NSCLC. *GJA4* is a gap junction (GJ) protein also known as *Cx37*. *GJs* are involved in intracellular communication through junctions and have an important role in homeostasis. The disruption of *GJs* results in pathological states, most commonly carcinogenesis (50, 51). The transmembrane proteins, connexins, form the gap junctions. Cxs can serve as tumor suppressors or tumor promoters depending on the stage and type of cancer (52).

*DOCK4* belongs to the *DOCK180* family, which has diverse cell- specific functions and plays an important role in the metastasis of various tumors such as breast cancer, melanoma, and glioblastoma. The proteins function by engaging in various protein-protein interactions. Yu et al. (53) investigated the role of *DOCK4* in the prometastatic effects of TGF-b in lung adenocarcinoma. Their study revealed that TGF-b induced rapid expression of DOCK4 in a Smad-dependent manner. The induction of *DOCK4* proved crucial in TGF-B-driven lung adenocarcinoma metastasis (53). *TBX2* (T-box) plays a pivotal role in embryonic development and the control of cell cycle progression and carcinogenesis. It has also been implicated in several cancers, including melanoma, pancreatic cancer, and breast cancer. Although studies have shown the role of *TBX2* as a tumor suppressor gene (54, 55), there is some evidence correlating the overexpression of *TBX2* in NSCLC. *TBX2* upregulation was found to be upregulated in NSCLC, making it an important prognostic marker in NSCLC (56). The *DDR* gene belongs to a novel class of receptor tyrosine kinases and has a potential role in cancer invasion. Evidence suggests that upregulation of *DDR1* in NSCLC contributes to progression and poor prognosis, resulting in increased invasiveness (57, 58). *CD24*, a ligand for P-selectin, has been shown to contribute to the metastatic capacity of CD24- expressing cells (59). Kristiansen et al. demonstrated the expression of *CD24* in NSCLC is an independent prognostic tumor marker, underscoring its importance in the metastatic progression of cancer (60). Several pieces of evidence have supported the role of hub genes in the progression and development of NSCLC. The study provides a better understanding of the genetic variations of the genes involved in cancer progression, in addition to their interaction with other proteins in the development of this disease.

D rug-gene network analysis has proven to be essential not only for understanding disease pathophysiology but also for the identification of new drug targets in drug design. The network has identified several potential candidate drugs that have shown associations with these genes.

## Conclusions

The study aids in sorting out disease-specific genetic variants from cDNA datasets using a network-based system- level approach. Several complex phenotypic mechanisms, such as cellular replication, apoptosis, mitotic division, and protein signaling, could be understood using this comprehensive and effective method. We have found significant genes (*SASH1, TBX2, HBEGF*, etc.) linked to NSCLC cancer that can serve as potential drug targets. Potential interactions of these genes with other essential genes leading to cell cycle progression and apoptosis, causing carcinogenesis, have been found. These results can unravel the possible mechanisms of NSCLC cancer progression and occurrence.

## Data availability statement

The datasets presented in this study can be found in online repositories. The names of the repository/repositories and accession number(s) can be found in the article/**Supplementary Material**.

## Author contributions

RA organized the database, RA and UI helped in manuscript writing and editing. AM and MS contributed to conception and design of study. All authors contributed to manuscript revision, read, and approved the submitted version.
