# Corrigendum: Identification and validation of differentially expressed genes for targeted therapy in NSCLC using integrated bioinformatics analysis

**DOI:** 10.3389/fonc.2024.1377858

**Published:** 2024-04-08

**Authors:** Reem Altaf, Umair Ilyas, Anmei Ma, Meiqi Shi

**Affiliations:** ^1^ Department of Pharmacy, Iqra University, Islamabad, Pakistan; ^2^ Department of Pharmaceutics, Riphah Institute of Pharmaceutical Sciences, Riphah International University, Islamabad, Pakistan; ^3^ Department of Medical Oncology, Jiangsu Cancer Hospital and Jiangsu Institute of Cancer Research and The Affiliated Cancer Hospital of Nanjing Medical University, Nanjing, China; ^4^ Department of Clinical Pharmacy, School of Basic Medicine and Clinical Pharmacy, China Pharmaceutical University, Nanjing, China

**Keywords:** NSCLC, mutational analysis, differentially expressed genes, microarray, bioinformatics

In the published article, there was an error in affiliation of Meiqi Shi. Instead of “^4^Department of Clinical Pharmacy, School of Basic Medicine and Clinical Pharmacy, China Pharmaceutical University, Nanjing, China”, it should be “^3^Department of Medical Oncology, Jiangsu Cancer Hospital and Jiangsu Institute of Cancer Research and the Affiliated Cancer Hospital of Nanjing Medical University, Nanjing, China”.

The authors apologize for this error and state that they do not change the scientific conclusions of the article in any way. The original article has been updated.

