# Supplementary material for: Identification and validation of differentially expressed genes for targeted therapy in NSCLC using integrated bioinformatics analysis
Source: Front Oncol. 2023 May 31;13:1206768. doi: 10.3389/fonc.2023.1206768 (PMC10264625; doi:10.3389/fonc.2023.1206768)
Supplement: Supplementary file 1 [file Table_1.docx]

| **Supplementary Table 1.** Software and Tools used in this study | | |
| --- | --- | --- |
|  | | |
| **Database/Software/Tools** | **Accessibility** | **Utility** |
| National Center for Biotechnology Information (NCBI) | <https://ncbi.nlm.nih.gov> | Source of datasets accessibiltiy and genomic information |
| STRING Database | [STRING: functional protein association networks (string-db.org)](https://string-db.org/) | For interactions of protiens |
| DAVID Bioinformatics Tool 6.8 | [DAVID Functional Annotation Bioinformatics Microarray Analysis (ncifcrf.gov)](https://david.ncifcrf.gov/) | Funationla Annotation and Gene ontology |
| FunRich version 3 | [FunRich :: Functional Enrichment Analysis Tool :: Home](http://www.funrich.org/) | Enrichment analysis, Protien interaction, miRNA prediction |
| Geo2R | [GEO2R - GEO - NCBI (nih.gov)](https://www.ncbi.nlm.nih.gov/geo/geo2r/) | Statisticcal computing/Data minning |
| Comparative Toxicogenomic DATABASE | <https://ctdbase.org/> | chemical-gene analysis |
| GEPIA | [GEPIA (Gene Expression Profiling Interactive Analysis) (cancer-pku.cn)](http://gepia.cancer-pku.cn/) | Validation of expression of genes |
| The Human Protein Atlas Database | [The Human Protein Atlas](https://www.proteinatlas.org/) | Expression of genes in tissue |
