# Supplementary material for: Identification and validation of differentially expressed genes for targeted therapy in NSCLC using integrated bioinformatics analysis
Source: Front Oncol. 2023 May 31;13:1206768. doi: 10.3389/fonc.2023.1206768 (PMC10264625; doi:10.3389/fonc.2023.1206768)
Supplement: Supplementary file 2 [file Table_2.docx]

**Supplementary Table 2**: Mutational visualization lot of each interactive protein mutation

| **Mutation** | **Position of mutation in Protein** | **Reference amino acid residue in protein** | **Mutated amino aicd residue in protein** | **Count** | **Impact on closest PTM site** | **Number of adjacent PTMs affected** | **Affected site** |
| --- | --- | --- | --- | --- | --- | --- | --- |
| SASH1 K364N | 364 | K | N | 1 | none | 0 |  |
| SASH1 G370W | 370 | G | W | 1 | distal | 1 | 374S |
| SASH1 G421C | 421 | G | C | 1 | distal | 1 | 417S |
| SASH1 R684S | 684 | R | S | 1 | none | 0 |  |
| SASH1 D841Y | 841 | D | Y | 1 | proximal | 2 | 837S 839S |
| SASH1 P925R | 925 | P | R | 1 | network-rewiring - motif gain | 3 | 920R 923S 932Y |
| SASH1 S1008R | 1008 | S | R | 1 | network-rewiring - motif gain | 1 | 1013S |
| SASH1 G1118V | 1118 | G | V | 1 | none | 0 |  |
| SASH1 R1158S | 1158 | R | S | 1 | none | 0 |  |
| SASH1 L1231R | 1231 | L | R | 1 | network-rewiring - motif gain | 1 | 1233S |
| TBX2 S170W | 170 | S | W | 1 | none | 0 |  |
| TBX2 M566R | 566 | M | R | 1 | none | 0 |  |
| TBX2 V667E | 667 | V | E | 1 | none | 0 |  |
| DDR1 L91V | 91 | L | V | 1 | none | 0 |  |
| DDR1 D171Y | 171 | D | Y | 1 | none | 0 |  |
| DDR1 K243N | 243 | K | N | 1 | none | 0 |  |
| DDR1 R330G | 330 | R | G | 1 | none | 0 |  |
| DDR1 C876F | 876 | C | F | 1 | none | 0 |  |
| DOCK4 Y61F | 61 | Y | F | 1 | none | 0 |  |
| DOCK4 E155Q | 155 | E | Q | 1 | none | 0 |  |
| DOCK4 P194L | 194 | P | L | 1 | none | 0 |  |
| DOCK4 P300A | 300 | P | A | 1 | none | 0 |  |
| DOCK4 V378L | 378 | V | L | 1 | distal | 1 | 375Y |
| DOCK4 R516K | 516 | R | K | 1 | none | 0 |  |
| DOCK4 P519L | 519 | P | L | 1 | none | 0 |  |
| DOCK4 E700D | 700 | E | D | 1 | none | 0 |  |
| DOCK4 G724C | 724 | G | C | 1 | none | 0 |  |
| DOCK4 T796S | 796 | T | S | 1 | none | 0 |  |
| DOCK4 N976S | 976 | N | S | 1 | none | 0 |  |
| DOCK4 G1056V | 1056 | G | V | 1 | none | 0 |  |
| DOCK4 S1100R | 1100 | S | R | 1 | none | 0 |  |
| DOCK4 L1380F | 1380 | L | F | 1 | none | 0 |  |
| DOCK4 E1522D | 1522 | E | D | 1 | none | 0 |  |
| DOCK4 L1770V | 1770 | L | V | 1 | proximal | 1 | 1769S |
| DOCK4 E1866K | 1866 | E | K | 1 | none | 0 |  |
| DOCK4 R1955L | 1955 | R | L | 1 | none | 0 |  |
| GATA3 G128W | 128 | G | W | 1 | none | 0 |  |
| GATA3 P157T | 157 | P | T | 1 | network-rewiring - motif loss | 2 | 156T 162S |
| GATA3 H200Q | 200 | H | Q | 1 | distal | 1 | 195K |
| GATA3 A268E | 268 | A | E | 1 | distal | 1 | 261R |
| GATA3 G278S | 278 | G | S | 1 | distal | 1 | 282Y |
| GATA3 A318S | 318 | A | S | 1 | none | 0 |  |
| GATA3 D335H | 335 | D | H | 1 | none | 0 |  |
| GATA3 M442I | 442 | M | I | 2 | none | 0 |  |
| GJA4 E53K | 53 | E | K | 1 | none | 0 |  |
| GJA4 G60C | 60 | G | C | 1 | none | 0 |  |
| GJA4 L231M | 231 | L | M | 1 | none | 0 |  |
| GJA4 R234H | 234 | R | H | 1 | none | 0 |  |
| HBEGF L26R | 26 | L | R | 1 | none | 0 |  |
| HBEGF T44I | 44 | T | I | 1 | direct | 2 | 44T 47T |
| HBEGF A82T | 82 | A | T | 1 | distal | 4 | 75T 77S 78S 85T |
| HBEGF L109V | 109 | L | V | 1 | none | 0 |  |
| HBEGF R128W | 128 | R | W | 1 | none | 0 |  |
| HBEGF D158G | 158 | D | G | 1 | none | 0 |  |
| HBEGF A164G | 164 | A | G | 1 | none | 0 |  |
| NPR1 M341L | 341 | M | L | 1 | none | 0 |  |
| NPR1 G418C | 418 | G | C | 1 | none | 0 |  |
| NPR1 Q431K | 431 | Q | K | 1 | none | 0 |  |
| NPR1 D678N | 678 | D | N | 1 | none | 0 |  |
| NPR1 P762H | 762 | P | H | 1 | none | 0 |  |
| NPR1 W784R | 784 | W | R | 1 | none | 0 |  |
| NPR1 R798C | 798 | R | C | 1 | none | 0 |  |
